# Queen Loss Remodels Brain Dopamine and Hormonal Pathways During Worker Ovary Activation in *Apis mellifera*

**DOI:** 10.3390/insects17030308

**Published:** 2026-03-12

**Authors:** Meijiao Zhao, Jiangli Wu, Weipeng Kang, Qiaohong Wei, Shufa Xu, Honggang Guo, Bin Han

**Affiliations:** 1 College of Bioscience and Resource Environment, Beijing University of Agriculture, Beijing 102206, China; 2 State Key Laboratory of Resource Insects, Institute of Apicultural Research, Chinese Academy of Agricultural Sciences, Beijing 100193, China

**Keywords:** *Apis mellifera*, worker fertility, dopamine, dopamine receptors, hormone signaling, ovary activation

## Abstract

In honey bees, reproductive division of labor is maintained by social suppression of worker fertility, yet queen loss can trigger ovary activation in workers. Here, we tested whether endogenous dopaminergic signaling is progressively remodeled across successive stages of ovarian activation and how these changes relate to key hormone pathways. Newly emerged *Apis mellifera* workers were introduced into queenright or queenless colonies, collected after 14 days, and classified as having inactive, partially activated, or fully activated ovaries. We quantified brain dopamine and measured expression levels of genes involved in dopamine synthesis, transport, metabolism, and reception in both brain and ovary tissues, together with transcriptional markers of juvenile hormone (JH) and 20-hydroxyecdysone (20E) signaling. Brain dopamine increased with ovary activation and peaked in fully activated workers, coincident with elevated transcripts of *tyrosine hydroxylase*, *dopa decarboxylase*, *dopamine transporter*, and *arylalkylamine N-acetyltransferase* in the brain. Dopamine receptor genes were stable in the brain but were remodeled in the ovary, with *Amdop1* increasing and *Amdop3* decreasing during activation. Markers of JH signaling and ovarian 20E pathway activity also rose with ovarian development, consistent with early endocrine priming following queen loss. Collectively, these results support an integrated neuroendocrine framework in which dopaminergic remodeling and hormone pathway activation jointly accompany worker reproductive activation under queenless conditions.

## Simple Summary

In honey bee colonies, a single queen usually produces most of the eggs, while worker bees typically do not reproduce. When the queen is lost, some workers can activate their ovaries and begin laying eggs, helping the colony persist. However, the biological signals that connect queen loss to worker reproduction are not fully understood. We studied worker bees from colonies with and without a queen and compared individuals at three stages of ovary development, from inactive to fully active. We found that workers with fully active ovaries had higher levels of dopamine, a chemical messenger in the brain. At the same time, key genes that support dopamine production and movement in the brain increased. In the ovary, two dopamine receptors shifted in opposite directions as ovaries became active, suggesting that the ovary’s sensitivity to dopamine is reorganized during reproduction. We also observed increased activity of two major insect hormone systems that regulate reproduction. Together, these results show how brain signaling, ovarian sensitivity, and hormone-related changes may work together to enable worker reproduction after queen loss.

## 1. Introduction

Reproductive division of labor is a defining feature of eusocial insects: in highly integrated colonies, queens specialize in reproduction, whereas most workers are functionally sterile [1]. This social organization secures the queen’s central reproductive role and enhances colony efficiency through worker specializations [2]. In honey bees (*Apis mellifera*), worker ovaries usually remain inactive in the presence of a queen, whereas queen loss can trigger ovarian activation in a subset of workers, enabling egg laying and partially buffering colony reproductive failure [3]. Understanding how social context is translated into endocrine and molecular changes within worker reproductive tissues is, therefore, central to explaining both the proximate regulation of worker fertility and the evolutionary stability of eusociality.

Worker reproduction in honey bees is regulated by multiple interacting mechanisms, including queen pheromones, worker–worker interactions, nutritional physiology, and endocrine signaling [4,5]. Queen mandibular pheromone (QMP) is a key inhibitory cue that suppresses worker ovary activation and reinforces reproductive skew [6,7]. Functional evidence indicates that canonical developmental pathways can be co-opted to maintain this constraint: chemical inhibition of Notch signaling, for example, can overcome the repressive effect of queen signals and promote ovary activity in adult workers [8]. At the endocrine level, juvenile hormone (JH) and ecdysteroids, particularly 20-hydroxyecdysone (20E), are major regulators of insect reproduction and behavior [9,10,11], and in bees, their effects are pleiotropic and strongly context dependent, varying with developmental stage and the degree of social organization [12]. In honey bees, 20E-responsive genes such as *Broad-Complex* and *E75* are expressed in both brain and ovary, consistent with active 20E signaling in tissues relevant to reproductive physiology [13]. Transcriptomic comparisons between activated and inactivated worker ovaries further support coordinated remodeling of endocrine, metabolic, and oogenesis-related programs during ovary activation [14]. Despite this progress, how neural and neuroendocrine signals, especially biogenic amines, interface with JH/20E axes during queenless ovary activation remains unresolved.

Among biogenic amines, dopamine has emerged as a particularly compelling candidate for mediating socially induced reproductive plasticity. Dopamine is classically studied as a neuromodulator of motivation, learning, and motor function [15,16,17,18], but in insects, it also participates in reproduction-associated physiology and caste-related transitions [19,20,21,22]. In honey bees, early work reported that brain dopamine levels are elevated in queenless workers with developing ovaries, suggesting a tight association between dopaminergic state and reproductive potential [23]. Subsequent experiments provided causal support: dietary dopamine supplementation increased the frequency of ovary activation in queenless workers, indicating that elevated dopamine can promote worker reproductive development under queen absence [24]. At the same time, the generality of dopamine’s gonadotropic role remains debated, as more recent work found no effect of dopamine supplementation on ovarian activity under certain experimental conditions [25]. These mixed outcomes underscore the need to move beyond single-factor supplementation tests and to interrogate the endogenous dynamics of dopaminergic signaling across defined stages of worker ovary activation.

Mechanistically, dopamine can influence reproduction through at least two non-mutually exclusive routes: (i) modulation of neural state and endocrine output, and (ii) direct action within peripheral tissues, including the ovary [26,27]. Social signals strongly implicate the first route. QMP modulates brain dopamine pathways in workers, affecting dopamine levels, dopamine receptor gene expression, and dopaminergic responsiveness, thereby providing a direct molecular entry point for queen regulation of worker neurochemistry [28]. Equally important, evidence supports tissue-localized dopaminergic signaling in the ovary. In worker honey bees, biogenic amine receptor genes, including dopamine receptors, are expressed in ovarian tissue, and their expression correlates with worker reproductive status, suggesting that dopamine may act directly on the ovary to regulate worker sterility and activation [29]. However, what remains largely unknown is how endogenous dopamine content, dopamine receptor expression, and the broader molecular machinery for dopamine synthesis, transport, and metabolism change in concert across progressive stages of ovary activation in queenless colonies.

A second major gap concerns the interaction between dopamine and canonical endocrine pathways. In insects, cross-talk among biogenic amines, JH, and 20E can be functionally significant and environmentally sensitive [30,31,32,33]. Evidence from *Drosophila* further indicates coordinated interactions among dopamine, JH, and 20E under normal and nutritional stress conditions, with consequences for reproductive output [34]. In bees, JH and 20E are recognized as major regulators of brain, behavior, and reproduction [12]. Yet, in honey bee workers, particularly during queenless ovary activation, the extent to which dopaminergic signaling aligns with, precedes, or potentially synergizes with JH/20E pathway activity remains poorly resolved at the molecular level. Addressing this question requires parallel profiling of dopaminergic components and endocrine pathway markers within the same biological framework of graded ovary activation.

Here, we leveraged worker honey bees (*Apis mellifera*) from queenless colonies representing discrete stages of ovary activation to systematically map dopaminergic dynamics and their links to JH and 20E signaling. We quantified brain dopamine levels and profiled the expression of dopamine receptors as well as core genes involved in dopamine synthesis, transport, and metabolism in workers with inactive, partially activated, and fully activated ovaries. In parallel, we measured transcriptional markers of JH biosynthesis and signaling, together with key components of the ovarian 20E pathway, to test whether dopaminergic variation was coordinated with endocrine remodeling during ovary activation. By integrating neurochemical measurements with tissue-specific expression profiles across graded activation states, this study provided a unified molecular view of dopaminergic engagement in worker reproductive activation under queenless conditions. Collectively, these findings offer a framework for dissecting how dopamine signaling intersects with JH and 20E pathways to shape worker fertility and motivate targeted functional tests in future work.

## 2. Materials and Methods

### 2.1. Honey Bees

All colonies used in this study were Western honey bees (*Apis mellifera*) maintained at the Xiangshan experimental apiary, Institute of Apicultural Research, Chinese Academy of Agricultural Sciences. Experiments were conducted from April to September 2024.

### 2.2. Experimental Design

Twelve healthy colonies were randomly assigned to three groups: a queenless treatment group (*n* = 3 colonies), a queenright control group (*n* = 3 colonies), and a queenright donor group (*n* = 6 colonies). Queenless colonies were established in early April by removing the resident queen. Each colony was standardized to five frames: two honey frames, one drone-brood frame, and two frames containing empty cells, pollen/nectar, and worker brood. Colonies were inspected weekly, and any emergency queen cells were removed. A colony was considered successfully queenless when multiple eggs appeared irregularly within individual cells, a hallmark of worker oviposition. Control colonies retained their queens and were standardized to the same frame composition as treatment colonies to maintain natural reproduction. Donor colonies were queenright and maintained under standard management.

Once worker oviposition was observed in queenless colonies, capped brood frames from donor colonies that were 1–2 days from adult emergence were incubated in a controlled chamber (34 °C, 60% ± 5% relative humidity). Newly emerged workers (1 day old; emerged within 12 h; *n* = 1000) were collected, marked on the thorax with distinct paint colors, and introduced into the treatment and control colonies. On day 14 after introduction, marked workers were collected from each colony and transferred to the laboratory on ice for dissection. Ovaries were dissected under a Leica EZ4W stereomicroscope (Leica Microsystems Ltd., Wetzlar, Germany) and classified into three developmental grades following a published scheme: (i) inactive (level 1), thin ovarioles with no visible oocytes; (ii) partially activated (level 2), elongated ovaries with a few ovarioles containing discernible oocytes; and (iii) fully activated (level 3), multiple ovarioles bearing well-developed oocytes, resembling queen-like ovaries [35].

After phenotyping, tissues were collected according to the ovary activation level. For the control group, only level 1 workers were sampled. For the queenless treatment group, workers from levels 1–3 were sampled. For each worker, the head, brain, and ovary were collected. Head samples used for RT-qPCR were prepared as whole-head homogenates after dissection and thus included endocrine tissues remaining within the head capsule; the corpora allata/corpora cardiaca complex was not specifically isolated or excluded. All tissues were placed into RNase-free tubes, snap-frozen in liquid nitrogen, and stored at −80 °C until analysis. Each biological replicate consisted of a pooled sample of workers collected from multiple colonies rather than from a single colony. Accordingly, the experimental unit for molecular and biochemical analyses was the pooled multi-colony worker sample.

### 2.3. Dopamine Quantification in the Brain

Brain dopamine content was measured using an Insect Dopamine (DA) ELISA kit (YJ301601; Yuanju Bio-Technique, Shanghai, China). Briefly, 20 mg of brain tissue was homogenized in 40 μL phosphate-buffered saline (PBS). Homogenates were centrifuged at 4000× *g* for 10 min at 4 °C, and the supernatant was used for ELISA.

Assays were performed according to the manufacturer’s instructions. Standard, sample, and blank wells were prepared. Standards (0, 7.5, 15, 30, 60, and 120 pg/mL) were added at 50 μL per well. For samples, 10 μL of supernatant plus 40 μL sample diluent were added per well. Blank wells received no sample. HRP-conjugated detection antibody (100 μL) was added to standard and sample wells, plates were sealed, and incubated at 37 °C for 60 min. Wells were washed five times. Substrates A and B (50 μL each) were added and incubated for 15 min at 37 °C in the dark. Stop solution (50 μL) was added, and absorbance was read at 450 nm within 15 min using a microplate reader (Multiskan GO, Thermo Scientific, Waltham, MA, USA). Dopamine concentrations were calculated from a standard curve.

Each treatment included three biological replicates; each biological replicate was measured in triplicate technical replicates and averaged.

### 2.4. Gene Expression Analysis

Total RNA was extracted from heads, brains, and ovaries using TRIzol reagent (three biological replicates per group; ~100 mg tissue per replicate). RNA quality and concentration were assessed prior to cDNA synthesis. First-strand cDNA was synthesized using All-in-One First-Strand Synthesis MasterMix (with dsDNase) (F0202; LABLEAD, Beijing, China).

Gene-specific primers were designed from NCBI reference sequences using Primer Premier 5.0. Targets included dopamine biosynthesis genes *tyrosine hydroxylase* (*Amth*) and *dopa decarboxylase* (*Amddc*); dopamine receptors (*Amdop1*, *Amdop2*, *Amdop3*); dopamine transporter (*Amdat*); dopamine metabolism gene *arylalkylamine N-acetyltransferase* (*Amnat*); juvenile hormone (JH) pathway genes *juvenile hormone acid O-methyltransferase* (*JHAMT*), *methyl farnesoate epoxidase* (*MFE*), the JH receptor *methoprene-tolerant* (*Met*), and the early-response transcription factor *Krüppel-homolog 1* (*Kr-h1*); and 20E pathway genes *Neverland* and *Shadow*, the *ecdysone receptor* (*EcR*), and the early-response gene *E75*. Primer sequences are provided in Table A1.

RT-qPCR was performed using 2× Realab Green PCR Fast mixture (R0202; LABLEAD, Beijing, China). Each 15 μL reaction contained 1 μL cDNA, 7.5 μL Taq SYBR^®^ Green qPCR Premix, 0.5 μL each of forward and reverse primers, and nuclease-free water to volume. Cycling conditions were: 95 °C for 30 s; 40 cycles of 95 °C for 10 s, 60 °C for 30 s. Among four candidate reference genes (*β-actin*, *RP49*, *Ef1a*, and *GAPDH*), *β-actin* and *GAPDH* were identified as the most stable using BestKeeper [36], and were used for normalization. Relative expression was calculated using the 2^−ΔΔCt^ method.

### 2.5. Statistical Analysis

All statistical analyses were performed in GraphPad Prism 10. Data normality was assessed using the Shapiro–Wilk test and homogeneity of variances using Levene’s test. When assumptions of normality and homoscedasticity were met, one-way ANOVA followed by Tukey’s post hoc test was applied. When variances were unequal, Welch’s ANOVA followed by the Games–Howell test was used. When parametric assumptions were not met, Kruskal–Wallis tests were performed. The significance threshold was set at *p* < 0.05.

## 3. Results

### 3.1. Brain Dopamine Levels Across Ovary Activation States

**Figure 1:**
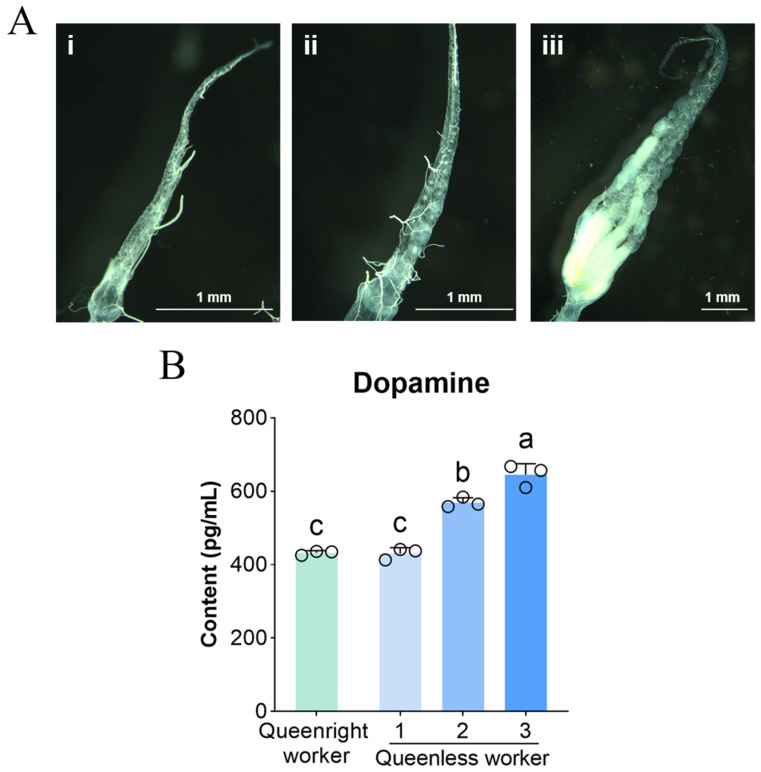
Ovarian activation phenotypes and brain dopamine levels in worker bees. (**A**) Representative ovarian morphologies corresponding to three activation states: (**i**) inactive ovaries, with thin ovarioles and no visible oocytes; (**ii**) partially activated ovaries, retaining a slender morphology but showing gaps along the ovariole and/or clearly visible oocytes in a few ovarioles under microscopy; and (**iii**) fully activated ovaries, with mature oocytes present in multiple ovarioles, resembling a queen-like ovarian phenotype. (**B**) Brain dopamine content in workers across ovarian activation states. Data are presented as mean ± SD (*n* = 3 biological replicates per group). Different letters indicate significant differences (one-way ANOVA with Tukey’s post hoc test, *p* < 0.05).

To examine whether dopamine is associated with worker ovary activation, we quantified brain dopamine content in workers with different ovary activation levels (Figure 1). Dopamine levels in level 1 workers did not differ between queenright and queenless colonies (*p* > 0.05). In contrast, within queenless colonies, dopamine content in fully activated workers (level 3) was significantly higher than in level 2 and level 1 workers (*p* < 0.05), reaching 1.50-fold and 1.11-fold of the latter groups, respectively. Dopamine levels in level 2 workers were also significantly higher than in level 1 workers (*p* < 0.05).

### 3.2. Expression of Dopamine Synthesis, Transport, and Metabolism Genes in the Brain

**Figure 2:**
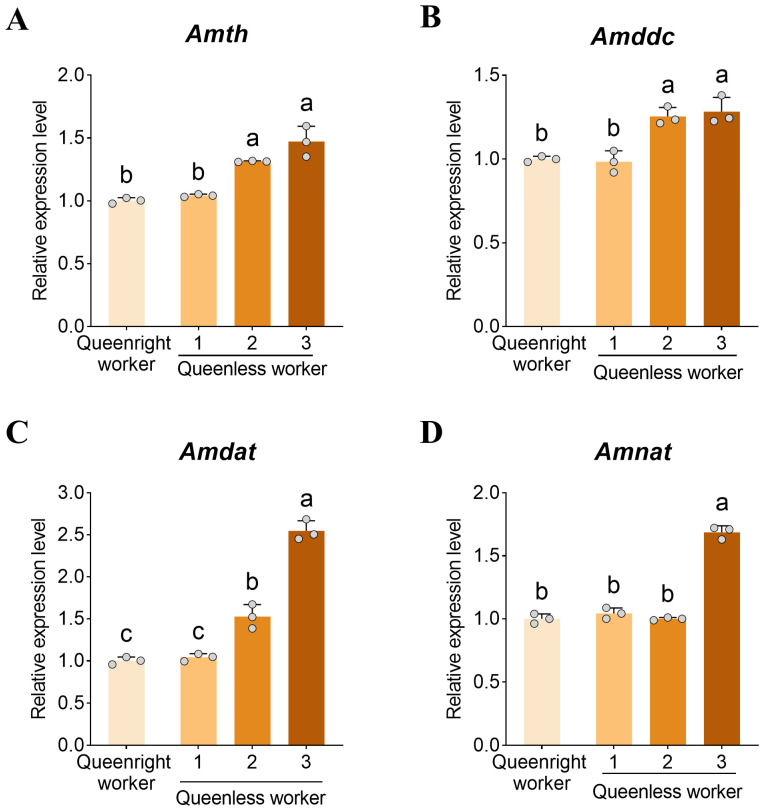
Ovarian activation–dependent changes in brain expression of dopamine synthesis, transport, and metabolism genes. Relative transcript levels of (**A**) dopamine biosynthesis gene *Amth* (*tyrosine hydroxylase*) and (**B**) *Amddc* (*dopa decarboxylase*), (**C**) dopamine transporter gene *Amdat*, and (**D**) dopamine metabolism gene *Amnat* (*arylalkylamine N-acetyltransferase*) in worker brains across ovarian activation states. Data are presented as mean ± SD (*n* = 3 biological replicates per group). Different letters indicate significant differences (one-way ANOVA with Tukey’s post hoc test, *p* < 0.05).

To investigate the molecular basis of the observed dopamine differences, we analyzed the expression of genes involved in dopamine synthesis, metabolism, and transport in the brain. *Amth* (Figure 2A) and *Amddc* (Figure 2B) showed similar expression patterns across ovary activation states. In level 1 workers, *Amth* and *Amddc* expression did not differ between queenright and queenless colonies (*p* > 0.05). Within queenless colonies, expression of *Amth* and *Amddc* was significantly upregulated in level 3 workers (*p* < 0.05), reaching 1.41-fold and 1.30-fold of level 1 workers, respectively, whereas the difference between levels 3 and 2 was not significant (*p* > 0.05).

The dopamine transporter gene *Amdat* was significantly upregulated in workers with activated ovaries (*p* < 0.05), with levels 3 and 2 reaching 2.44-fold and 1.67-fold of level 1 (Figure 2C). The dopamine metabolism gene *Amnat* was also expressed at significantly higher levels in level 3 brains compared with all other groups (all *p* < 0.05, Figure 2D).

### 3.3. Expression of Dopamine Receptor Genes in the Brain and Ovary

**Figure 3:**
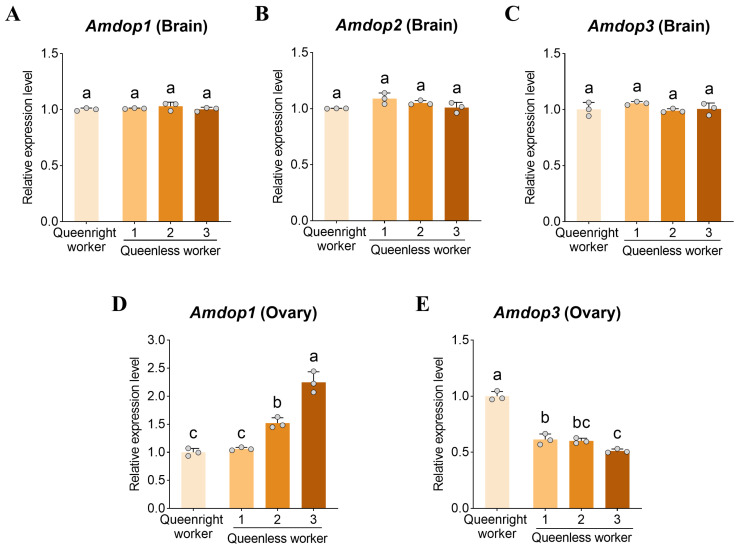
Dopamine receptor gene expression in the brain and ovary across worker ovarian activation states. Relative transcript levels of dopamine receptor genes in worker (**A**–**C**) brains and (**D**,**E**) ovaries at inactive, partially activated, and fully activated ovarian stages. Data are presented as mean ± SD (*n* = 3 biological replicates per group). Different letters indicate significant differences (one-way ANOVA with Tukey’s post hoc test, *p* < 0.05).

To further evaluate the potential role of dopamine signaling in worker reproduction, we quantified dopamine receptor gene expression in both the brain and the ovary. *Amdop1* and *Amdop3* were detected in both tissues, whereas *Amdop2* was detected in the brain but not in the ovary. In the brain, expression of *Amdop1*, *Amdop2*, and *Amdop3* did not differ significantly among workers with different ovary activation states (all *p* > 0.05, Figure 3A–C).

In the ovary, *Amdop1* expression was positively associated with ovary activation (Figure 3D). *Amdop1* was significantly higher in level 3 ovaries than in other groups (*p* < 0.05), reaching 2.12-fold of level 1. In contrast, *Amdop3* expression was highest in queenright workers (Figure 3E), being 1.96-fold higher than in level 3 workers from queenless colonies (*p* < 0.05). Within queenless colonies, *Amdop3* expression in level 1 workers was 1.20-fold that of level 3 workers (*p* < 0.05), whereas other pairwise comparisons were not significant (*p* > 0.05).

### 3.4. Expression of JH Signaling Genes

**Figure 4:**
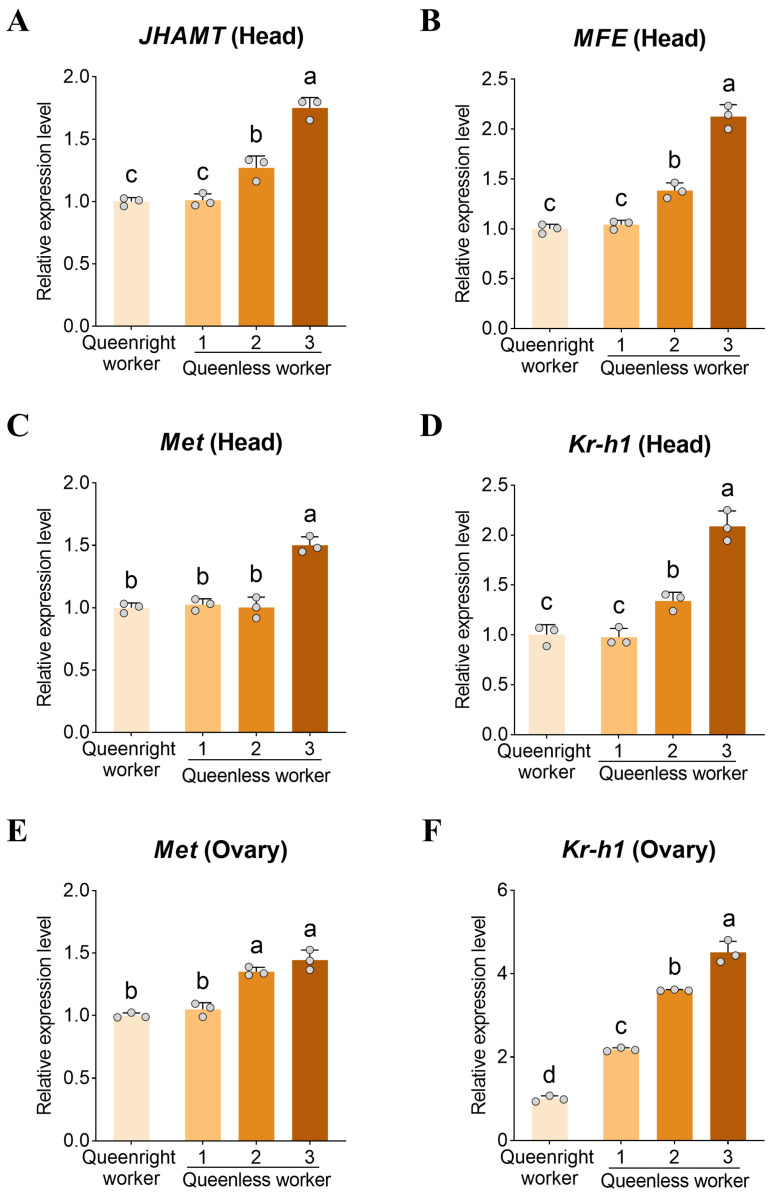
Juvenile hormone pathway gene expression in the head and ovary across worker ovarian activation states. Relative transcript levels of juvenile hormone (JH) pathway genes in worker (**A**–**D**) heads and (**E**,**F**) ovaries across ovarian activation states. Genes assayed include JH biosynthesis genes (**A**) *JHAMT* (*juvenile hormone acid O-methyltransferase*) and (**B**) *MFE* (*methyl farnesoate epoxidase*), the JH receptor (**C**,**E**) *Met* (*methoprene-tolerant*), and the early-response transcription factor (**D**,**F**) *Kr-h1* (*Krüppel-homolog 1*). Data are presented as mean ± SD (*n* = 3 biological replicates per group). Different letters indicate significant differences (one-way ANOVA with Tukey’s post hoc test, *p* < 0.05).

To assess potential links between dopamine and JH signaling, we quantified the expression of *JHAMT*, *MFE*, *Met*, and *Kr-h1* in the head and ovary. In head tissue, *JHAMT* and *MFE* expression increased significantly with the degree of ovary activation (Figure 4A,B) and peaked in level 3 workers (*p* < 0.05). Head expression of *Met* and *Kr-h1* was also significantly higher in level 3 workers than in other groups (*p* < 0.05, Figure 4C,D). *Kr-h1* expression was significantly higher in level 2 than in level 1 workers (*p* < 0.05), whereas *Met* did not differ between these two groups (*p* > 0.05).

In ovaries, *Met* and *Kr-h1* expression was significantly higher in activated groups (level 2 and 3 workers) than in inactive ovaries (level 1) (*p* < 0.05, Figure 4E,F). *Kr-h1* expression likewise increased with ovary activation; notably, *Kr-h1* expression in level 1 ovaries from queenless colonies was significantly higher than in level 1 ovaries from queenright colonies (*p* < 0.05).

### 3.5. Expression of 20E Pathway Genes in the Ovary

**Figure 5:**
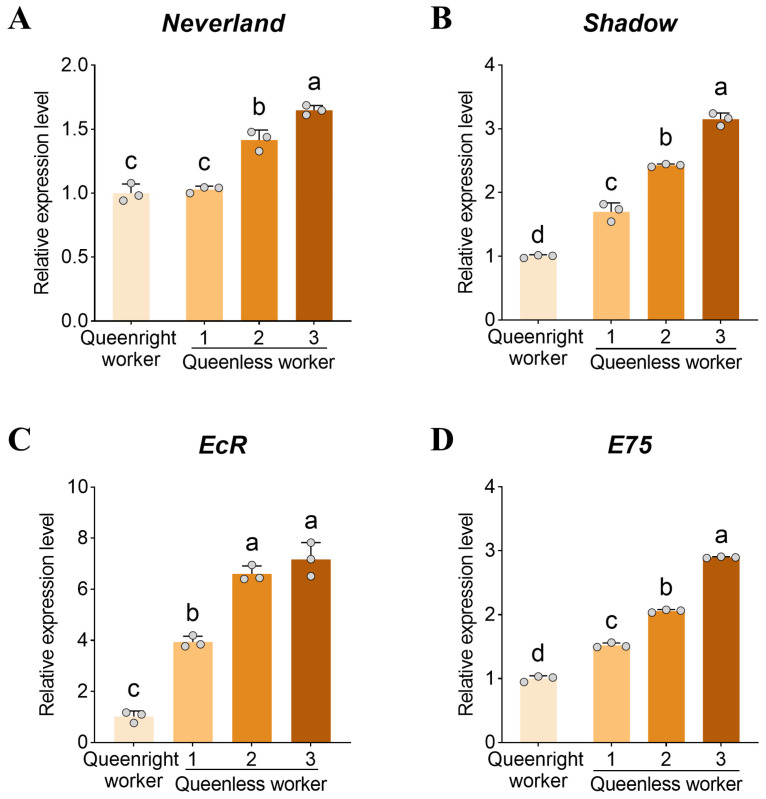
Ovarian activation–associated changes in 20-hydroxyecdysone pathway gene expression in worker ovaries. Relative transcript levels of 20-hydroxyecdysone (20E) pathway genes across ovarian activation states, including (**A**) *Neverland*, (**B**) *Shadow*, (**C**) the ecdysone receptor *EcR*, and (**D**) the early-response gene *E75*. Data are presented as mean ± SD (*n* = 3 biological replicates per group). Different letters indicate significant differences (one-way ANOVA with Tukey’s post hoc test, *p* < 0.05).

To examine associations between dopamine signaling and 20E signaling, we quantified the expression of *Neverland*, *Shadow*, *EcR*, and *E75* in ovaries. Expression of *Neverland*, *Shadow*, and *E75* increased significantly with ovary activation (*p* < 0.05, Figure 5A–D). *EcR* expression was significantly higher in levels 2 and 3 workers than in level 1 (*p* < 0.05), whereas levels 2 and 3 workers did not differ (*p* > 0.05, Figure 5C). Notably, in level 1 workers, ovarian expression of *Shadow*, *EcR*, and *E75* was significantly higher in queenless colonies than in queenright colonies (all *p* < 0.05, Figure 5B–D).

## 4. Discussion

In eusocial insects, worker sterility is not a fixed developmental endpoint but a socially regulated physiological state [37,38]. Our stage-resolved analysis identifies a coordinated neuroendocrine signature of worker reproductive activation in queenless *Apis mellifera* colonies. Three findings are central. First, workers with fully activated ovaries exhibit elevated brain dopamine together with concerted upregulation of dopamine synthesis (*Amth*, *Amddc*), transport (*Amdat*), and metabolism (*Amnat*), indicating a shift in dopaminergic capacity rather than a single-gene change. Second, ovarian activation was associated with a receptor rebalancing in the ovary—higher *Amdop1* and lower *Amdop3*—while brain dopamine receptor transcript levels remain comparatively stable. Third, transcriptional markers of JH and 20E pathway activity increased with activation and show evidence of early ovarian endocrine “priming” after queen loss. Collectively, these data are consistent with a model in which dopaminergic remodeling forms part of a central–peripheral neuroendocrine interface associated with endocrine remodeling and ovarian competence.

### 4.1. Central Dopaminergic Activation Is Coupled to Reproductive Commitment

Elevated brain dopamine has repeatedly been linked to the onset of worker fertility in queenless contexts. Early work showed that queenless workers with developing ovaries have higher dopamine in the brain [23], and dopamine supplementation can increase ovary activation under some conditions [24]. Our results strengthen these associations by demonstrating a graded relationship between brain dopamine and ovary activation state, suggesting that dopaminergic tone is coupled to the intensity or stability of reproductive commitment rather than simply reflecting queen absence.

A key advance of our dataset is that the neurochemical difference is supported by coordinated shifts across multiple pathway components. Upregulation of *Amth* and *Amddc* is consistent with increased dopamine synthesis capacity, while elevated *Amdat* suggests increased dopamine flux and recycling, not merely accumulation. The honey bee dopamine transporter is expressed in the brain and has been proposed to reflect dopaminergic neuron activity [39], and transporter variation has been linked to broader features of dopaminergic regulation in honey bees [40]. The increase in *Amnat* further implies that dopamine turnover is actively tuned during activation; insect arylalkylamine N-acetyltransferases are implicated in physiological switching and biogenic amine processing [41]. Together, these changes indicate a coordinated remodeling of dopamine production, clearance, and metabolism in reproductively activated workers.

A recent report found that dietary dopamine or L-DOPA did not alter ovarian activity in caged workers with or without queen cues [25]. Our results help reconcile such outcomes by implying that dopamine is embedded in a broader network: exogenous dopamine may not override endocrine or social gating when the physiological background is non-permissive. In this view, dopamine is more likely to contribute to crossing, reinforcing, or maintaining the activated state once other constraints (nutrition, colony demography, pheromonal complexity) are met, rather than serving as a single sufficient trigger.

### 4.2. Ovary-Specific Dopamine Receptor Rebalancing Provides a Mechanistic Link to Queen Regulation

The most mechanistically informative result is the ovary-specific shift in dopamine receptor expression, with *Amdop1* increasing and *Amdop3* decreasing during activation. This pattern is consistent with, yet goes beyond, prior evidence that dopamine receptor expression in the ovary is socially regulated. It has been shown that only *Amdop1* and *Amdop3* are detectable in worker ovaries and that queenless is associated with reduced *Amdop3*, with further reduction in queenless workers exhibiting activated ovaries [29]. Our stage-based design indicates that the activated ovary is characterized not only by low *Amdop3* but also by a relative upweighting of *Amdop1*, supporting a receptor-level “rewiring” of ovarian dopaminergic responsiveness.

Functional characterization of DOP3 provides a plausible interpretation for why *Amdop3* downregulation may be permissive for oogenesis. DOP3 is a D2-like dopamine receptor, and in heterologous expression assays, dopamine activation of *Am*DOP3 receptors produces inhibitory signaling consistent with reduced intracellular cAMP, a canonical D2-like output [42]. In contrast, D1-like receptors such as DOP1 are typically associated with cAMP elevation [43,44]. Although receptor coupling can be context dependent, the simplest inference is that increasing *Amdop1* while decreasing *Amdop3* would bias ovarian dopamine signaling toward a more activation-permissive second-messenger regime. This receptor rebalancing therefore suggests a testable mechanism by which dopamine could exert peripheral effects on ovarian physiology (e.g., follicle cell function, oocyte growth, or responsiveness to endocrine cues), independent of changes in brain receptor transcript abundance.

This framework also aligns with how queen pheromone influences dopaminergic pathways. Queen mandibular pheromone (QMP) modulates brain dopamine function in workers, and a QMP component, homovanillyl alcohol (HVA), has been implicated in dopaminergic modulation [28]. It has been proposed that *Amdop3* may participate in queen-associated suppression of worker reproduction and discussed the possibility that QMP-related components could engage *Amdop3* signaling [29]. While our study does not directly test QMP–receptor causality, the combined evidence supports a coherent model: queen presence maintains a high-*Amdop3* ovarian receptor state consistent with reproductive restraint, whereas queen loss is associated with disengagement of the *Amdop3* (D2-like) axis and increased reliance on *Amdop1*, thereby shifting ovarian competence toward activation.

### 4.3. Coordination with JH and 20E Pathways Suggests Endocrine Priming and Integrated Control

We observed parallel increases in transcriptional markers of JH biosynthesis/signaling (head *JHAMT*, *MFE*, *Met*, *Kr-h1*; ovarian *Met*, *Kr-h1*) and ovarian 20E biosynthesis/signaling (*Neverland*, *Shadow*, *EcR*, *E75*) with ovary activation. In bees, JH and 20E are major regulators of physiology and behavior with context-dependent roles in workers [12]. 20E-responsive genes such as *E75* are expressed in adult honey bee ovaries and brain [13], and transcriptome-scale comparisons show that ovary activation involves broad remodeling across endocrine and oogenesis-related programs [14]. Our contribution is to position dopaminergic remodeling within this endocrine landscape, showing that dopaminergic and hormone-pathway signatures rise together across defined activation stages.

One result of particular interest is that several ovarian 20E markers were already elevated in inactive workers from queenless colonies relative to queenright controls. This pattern suggests early endocrine priming after queen loss—i.e., an increase in ovarian endocrine competence before overt activation is morphologically apparent. Such priming could help explain why queenlessness does not uniformly induce full activation: the colony-wide endocrine state may shift, but only a subset of workers crosses the threshold into sustained oogenesis depending on additional constraints. This interpretation is consistent with the broader principle that hormone–neuromodulator interactions can be condition-dependent and responsive to environmental or social stressors [34]. At the same time, several limitations of the present study should be acknowledged. Dopamine was quantified using a commercial ELISA kit rather than a chromatographic method such as HPLC or LC–MS; accordingly, potential cross-reactivity or matrix effects should be considered when interpreting absolute dopamine values. Moreover, each biological replicate consisted of a pooled sample of workers collected from multiple colonies, and colony identity was therefore not incorporated as a random factor in the statistical analyses. Given the colony-structured nature of honey bee societies, this design does not allow colony-level variation to be explicitly resolved. Even so, because all samples were collected and processed consistently across groups, the data remain informative for relative comparisons among ovary activation states within the present experimental framework.

## 5. Conclusions

In summary, our staged analysis supports a cohesive molecular picture in which queen loss is associated with increased central dopaminergic tone, ovary-specific reconfiguration of dopamine receptor expression, and coordinated activation of JH and 20E pathway markers. These findings strengthen the hypothesis that dopamine participates in the neuroendocrine control of worker fertility, not as a solitary trigger but as part of an integrated network associated with endocrine remodeling and ovarian responsiveness. By establishing where in the dopaminergic pathway (synthesis, transport, metabolism, and receptors) changes occur and how these changes align with hormone signatures, our study identifies tractable targets for functional validation in future work.

## Data Availability

The original contributions presented in this study are included in the article. Further inquiries can be directed toward the corresponding authors.

## Appendix A

**Table A1:** Primers used in RT-qPCR.

Gene Symbol	NCBI Gene ID	Sequence (5′-3′)	Source
*Amdop1*	406111	F: GTGGTTGCTCGCAGGACTC R: GCGTAGGTGGGTGTAAGAT	This study
*Amdop2*	406070	F: TCGTAGAGCGTTCGTGAG R: AGGGAATAAGCGGAGTAG	This study
*Amdop3*	408995	F: CCCCTTGATCCTGTTGTG R: TCGTATCCCGAATCCTTT	This study
*Amth*	408930	F: GAGACCGAGACTTGGACC R: AAGAACTCGCTCACCTCC	This study
*Amddc*	410638	F: GAGACCGAGACTTGGACC R: TCAAGAACTCGCTCACCT	This study
*Amnat*	409361	F: GATCCTCCGAATGACGAAGA R: CGCTTTGGCAACTCCTTTAC	[45]
*Amdat*	412285	F: TGGCTACTCGATGCTGTTTG R: TATCGGAGCAACGAATTTCC	[45]
*JHAMT*	724216	F: ACGTGAAAGCCAGCACGATA R: GGTCCGCAACCTATGTCCAA	[46]
*MFE*	551632	F: TGATGGTAGACCAAGTGGATTT R: TGGCTATGCCAAACATCAGTAT	[46]
*Met*	411534	F: CAACATTTACCTCCTGCTGAAG R: GATCTCGTGTTTTCTTGTCTCTC	[46]
*Kr-h1*	100576395	F: GCATTGGAAGCAGTTGAAGAAG R: GAAGGTACAGGACTCACAGGAT	[46]
*Neverland*	410495	F: CGTTCCTCATGCCTCTTCACCAC R: ACCACGTCGTTCCAACTGTTCAC	[46]
*Shadow*	411893	F: CGCATCGCAGACATCAAGAATCG R: ACTTTCGCTAGTTGCTACCGCTAC	[46]
*EcR*	406084	F: CCTGGCTCTTTGAACGGGTATGG R: CCTCCTCCTCCTCCTCCTCCTC	[46]
*E75*	410309	F: ACGAGTAGTACGAGCACGAGTCC R: ACTGTCCGCTATGTCCACCTGTAG	[46]
*β-actin*	406122	F: CACTATACGCTTCTGGAC R: CTTTCTGTAAGGATCTTCATG	[46]
*GAPDH*	410122	F: CACCTTCTGCAAAATTATGGCG R: ACCTTTGCCAAGTCTAACTGTTAA	This study
